# Association of Statin Use with the Risk of Incident Prostate Cancer: A Meta-Analysis and Systematic Review

**DOI:** 10.1155/2022/7827821

**Published:** 2022-12-13

**Authors:** Meng-Yao Xu, Ye An, Chen-Qian Liu, Jin-Zhou Xu, Xing-Yu Zhong, Na Zeng, Jian-Xuan Sun, Qi-Dong Xia, Shao-Gang Wang

**Affiliations:** Department and Institute of Urology, Tongji Hospital, Tongji Medical College, Huazhong University of Science and Technology, No. 1095 Jiefang Avenue, Wuhan 430030, China

## Abstract

**Background:**

With the growth and aging of population, the incidence of prostate cancer will increase year by year, which is bound to bring greater economic burden to the society. There has been greater interest in the anticancer effects of statin in recent years. It is controversial whether statin use is associated with the risk of prostate cancer (PCa). Thus, we conducted a meta-analysis and systematic review to explore the effects of statin use and their duration and cumulative dose on the overall incidence of PCa.

**Method:**

The study was conducted according to the latest guidelines for PRISMA 2020. We searched PubMed and other databases for studies about the association of statin use with the risk of incident prostate cancer between January 1, 1990, and April 11, 2022. Two independent researchers extracted data and evaluated the quality of the studies. R x64 4.1.2 and random-effects model were used for data statistics. Relative risk (RR) and odds ratio (OR) effective values with a 95% confidence interval (95% CI) were used to assess the main results.

**Results:**

The results of 6 RCT and 26 cohort studies showed that statins did not significantly associate with the incidence of PCa (RR = 0.94, 95% CI: 0.82–1.08). The similar results were obtained from 9 case-control studies (OR = 1.03, 95% CI: 0.99–1.07). However, statins were associated with a lower risk of Pca (RR = 0.44, 95% CI: 0.28–0.70) when the cumulative defined daily dose (cDDD) was high. Using statins for more than five years could be associated with a reduced incidence of Pca (RR = 0.47, 95% CI: 0.23–0.97). There was a significant heterogeneity in these studies (RCT and cohort study: *I*^2^ = 98%, *P* < 0.01; case-control study: *I*^2^ = 72%, *P* < 0.01).

**Conclusion:**

We concluded that statins had a neutral association with the overall risk of PCa. High cDDD and long duration were associated with a lower risk of PCa.

## 1. Introduction

Prostate cancer (PCa) is the second most commonly diagnosed cancer and the sixth leading cause of cancer death in men, with an estimated 1.4 million diagnoses worldwide in 2020. With the growth and aging of the population, it is expected that the burden of PCa will grow to 2.3 million new cases and 740,000 deaths by 2040 [1]. The incidence is rising in many countries, while the mortality rate is declining in developed countries and rising in developing countries [2]. As the most populous country in the world, the incidence and mortality of PCa in China have increased with age from 40 to 94 years old in the past three decades [3]. Without intervention, PCa will bring a heavy burden to society. On the one hand, preventive measures can be taken to decrease the incidence of PCa. On the other hand, we can improve the diagnosis and treatment methods to decrease the mortality of PCa. In order to alleviate the pain of patients after diagnosis, we urgently need to find the protective factors of PCA.

Statins are 3-hydroxy-3-methylglutaryl-CoA (HMG-CoA) reductase inhibitors, which are comprehensive lipid-regulating agents. It can not only effectively decrease total cholesterol (TC) and low-density lipoprotein (LDL) but also decrease triglyceride (TG) and increase high-density lipoprotein (HDL). The mechanism of statins is that the intracellular cholesterol synthesis is decreased by competitively inhibiting the endogenous cholesterol synthesis rate-limiting enzyme (HMG-CoA reductase) and blocking the intracellular hydroxyvalerate metabolism pathway. Statins have been shown to work through mevalonate-dependent and mevalonate-independent mechanisms [4]. There has been greater interest in the anticancer effects of statins in recent years. Several studies have demonstrated an increased incidence of high-risk PCa in patients with elevated TC. Statins exert their anticancer effects by regulating patients' blood lipids [5]. Lee et al. [6] reported that one mechanism by which PCa cells maintain elevated intracellular cholesterol levels is hypermethylation of the *ABCA1* promoter and subsequent transcriptional silencing. In addition, a study [7] has shown that the accumulation of cholesterol esters in PCa cells results from the deletion of the tumor suppressor gene PTEN and the activation of the PI3K/AKT pathway. Since cholesterol is the precursor of androgen, cholesterol levels may also affect the development of PCa through androgen signaling pathways [8]. Therefore, statins decrease cholesterol levels through different mechanisms and have an anti-PCa effect.

More than 100 articles have reported that statin usage is associated with PCa, including the overall incidence of PCa and the risk of local or advanced PCa and so on. A previous meta-analysis [9] that included 6 randomized clinical trials, 6 cohort studies, and 7 case-control studies published in 2008 showed that statin usage did not decrease the overall risk of PCa. The other meta-analysis [10] included 15 cohort studies and 12 case-control studies published in 2012 showed that statin usage associated with a lower risk of overall PCa and clinically advanced PCa3. Another meta-analysis [11]that included 6 randomized clinical trials, 7 cohort studies, and 6 case-control studies published in 2017 revealed that neither hydrophilic nor hydrophobic statins were associated with the incidence of PCa. Most recent meta-analysis published in 2021 [12] that included 10 cohort studies and 4 case-control studies indicated that statin usage was associated with a lower risk of advanced PCa.

Thus, it is controversial whether statin usage is associated with the risk of PCa in different populations. Furthermore, the previous meta-analysis cannot well claim the effects of statins on prostate cancer that they did not include enough studies or their result analysis was incomplete. This meta-analysis and systematic review are a revision and update of all the previous clinical studies, focusing more on and aiming to unravel the effects of different types of statins and their duration and cumulative dose on the overall incidence rate of PCa.

## 2. Materials and Methods

This systematic review and meta-analysis were reported according to the latest guidelines for PRISMA 2020 (Supplementary Material 1).

### 2.1. Inclusion and Exclusion Criteria

All studies or literature included in the meta-analysis and systematic review strictly met the following PICOS (patients, interventions, comparators, outcomes, and study design) criteria:

1. *Patients*. Patients who were diagnosed with PCa were included.
2. *Interventions*. According to reliable records, there was a history of using statins.
3. *Comparators*. For RCT and cohort studies, the control group was participants who were not treated with statins. For case-control study, the control group was participants who was not diagnosed with PCa.
4. *Outcome*. In RCT and cohort studies, the participants number of exposed and nonexposed groups and their corresponding number of PCa patients need to be recorded to subsequently obtain relative risk (RR) with 95% confidence intervals (CIs). As for case-control studies, the participants number of case group and control group and the number of statin users and non-users in the two groups also need to be recorded, respectively, to get odds ratio (OR) with 95% confidence intervals (CIs).
5. *Study Design.* It included randomized controlled trials (RCTs) and cohort study and case-control study.

### 2.2. Literature Search

Two independent researchers (M-YX and J-XS) searched PubMed, Embase-Ovid, Cochrane Library, and ClinicalTrias.gov for publications between January 1, 1990, and April 11, 2022. We also searched some grey literature in Google Academic, American Society of Clinical Oncology (ASCO) meeting summary, European Urology Society (EAU) meeting summary, and American Urology Society (AUA) meeting summary. We used keywords to search for “Statin(s)” or “Atorvastatin” or “Cerivastatin” or “Compactin” or “Fluvastatin” or “HMG-CoA” or “Lovastatin” or “Mevastatin” or “Pravastatin” or “Rosuvastatin” or “Rosvastatin” or “Simvastatin” and “PCa” or “Prostate Neoplasms” and so on. Supplementary Material 2 shows the detailed search strategies for each database. After deleting duplicated data by EndNoteX9, the search results were sorted out based on the inclusion and exclusion criteria. We use the PRISMA flowchart to describe the literature search process.

### 2.3. Data Extraction

Two independent researchers (M-YX and J-XS) extracted data using a data extraction sheet that included basic information and some research factors of interest [13]. Basic information consisted of the author's name, year of publication, country, study design, and patient characteristics. We also recorded the follow-up period, age, body mass index (BMI), cholesterol, race (the percentage of white), the level of PSA, Gleason Score (GS), tumor stage, the definition of statin use, the number of patients, the number of statin users, the number of patients diagnosed with PCa, type of statins, cumulative duration, and cumulative defined daily dose (cDDD) (the cumulative total amount of DDD over time). In addition, the data about outcomes needed to be extracted, such as the adjusted multivariate relative risk (RR) or odds ratio (OR) with 95% confidence intervals (CIs).

### 2.4. Quality Assessment

Two researchers (M-YX and J-XS) independently assessed the quality of cohort and case-control studies using the Newcastle-Ottawa scale (NOS). The NOS scale which includes eight items consists of three parts (selection, comparability, and outcome). A score of 7 to 9 is defined as high quality, and a score less than 7 is defined as low quality.

We adapted RoB2 Excel to evaluate the quality of RCTs. RoB2 set up five evaluation domains: the randomization process, deviations from intended interventions, missing outcome data, measurement of the outcome, and selection of the reported result. There are many different signal problems in each domain. Researchers need to make judgments and answer these questions objectively when evaluating the bias risk of an RCT. There are generally five answers to signal questions: yes (Y), probably yes (PY), probably no (PN), no (N), and no information (NI). According to the reviewers' answers to the signal questions, the bias risk in each domain can be divided into three levels: “low risk,” “certain risk,” and “high risk.” Any differences are resolved by re-evaluating the original article with the third researcher.

### 2.5. Statistical Analysis

In RCT and cohort studies, the estimated RR effect values with 95% CI were used to assess the potential association between statin usage and PCa risk, whereas the OR effect values were used in case-control studies. We used Cochrane's *Q* and *I*^2^ statistics to test the heterogeneity. The random-effects model should be adopted due to expected heterogeneity among different study populations. In order to verify the publication bias of studies, we conducted Begg's and Egger's tests [14]. We conducted subgroup analyses of countries and statins type to find potential sources of heterogeneity. The difference between studies may be related to age, BMI, race (the percentage of white) (the percentage of white) and cDDD (the percentage of statin users whose cDDD >600), so we conducted meta-regression analysis to test this hypothesis. In addition, we performed sensitivity analysis and cumulative meta-analysis to estimate the stability of our meta-analysis by gradually omitting or adding included studies.

We used the “meta” package in R x64 4.1.2 to perform meta-analysis [15, 16]. All the statistical tests were bilateral, and the *P* value <0.05 indicated statistical significance.

## 3. Result

### 3.1. Search Results and Study Characteristics

**Figure 1:**
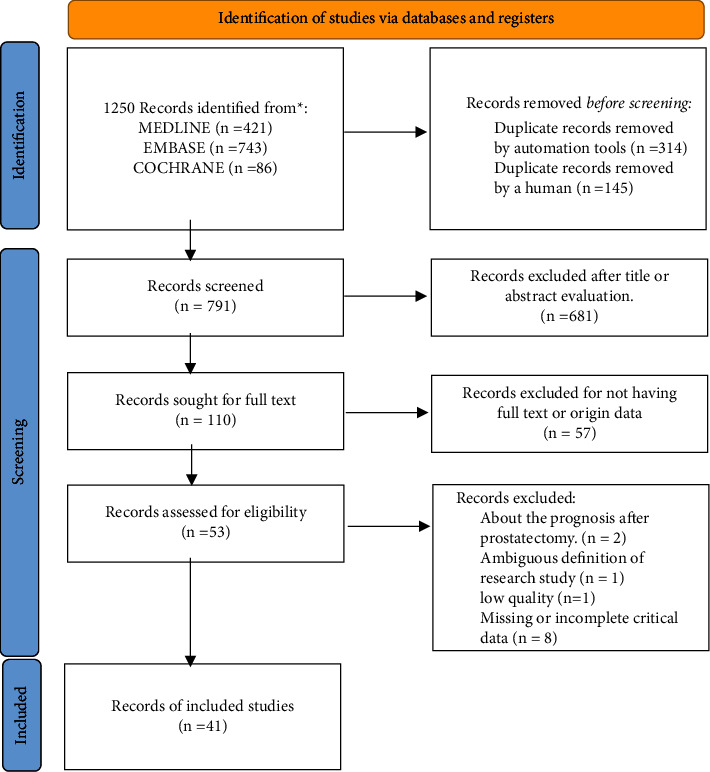
PRISMA (preferred reporting items for systematic reviews and meta-analyses) flow chart of studies selection.

We have retrieved 1,250 publications from electronic databases and grey literature. We remained 731 after removing repetitive literature. After a preliminary reading of the title and abstract, we retained 110 articles. After screening according to strict criteria, 57 articles were excluded. Then, we read the full text of each record. After excluding 12 publications due to a lack of data about the incidence of PCa, 41 studies were finally included in this meta-analysis and systematic review (Figure 1).

The characteristics of all studies included are shown in Supplementary Material 3. The literature included in this study was published from 1998 to 2022. This study included 6 randomized controlled trials [17–22], 26 cohort studies [23–48], and 9 case-control studies [49–57] which included a total of 3,697,172 participants. There was a total of 12 countries (Israel, Scotland, Australia, Canada, China Taiwan, Denmark, Finland, Japan, the Netherlands, Sweden, UK, and USA) in this meta-analysis. The top three countries with the most studies were the United States, Finland, and the United Kingdom, with 20, 5, and 3 studies, respectively. Participants used hydrophilic and hydrophobic statins, including fluvastatin, pravastatin, rosuvastatin, atorvastatin, cerivastatin, simvastatin, and lovastatin. It should be noted that “statin usage” was defined differently in different studies. Some studies had shown that participants could be regarded as statin users as long as they conducted a self-report or kept a record of drug purchases to prove that they had used statins. In other studies, researchers defined statin users as participants who used statins with a clear type, dose, and time.

### 3.2. Quality Assessment of Included Studies

As for the quality of the included studies, more details are presented in Tables 1 and 2. NOS was used to access the quality of 36 observational studies, and the scores of the included studies ranged from 5 to 9. The bias risk map and bias risk summary map (Supplementary Material 4) in ROB2 excel vividly demonstrated the literature quality of 6 RCT studies [58]. Except for some concerns about the selection of the reported result and some high-risk aspects of the measurement of the outcome, the other three domains were low risk. Furthermore, overall bias was low risk. However, among the included studies, the case-control study had the lowest evidence grade [59].

### 3.3. Statin Use and the Risk of PCa

**Figure 2:**
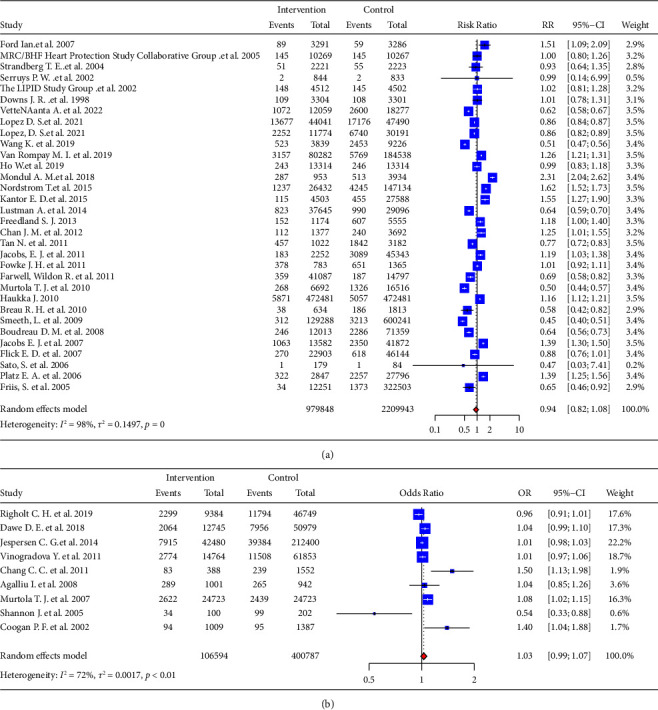
The effect of statin usage on the risk of incident prostate cancer using the random-effects model. (a) The forest plot for the RR. (b) The forest plot for the OR.

6 RCT studies and 26 cohort studies showed that statin use was not significantly associated with reducing the incidence of PCa (RR = 0.94, 95% CI: 0.82–1.08). Similar results were obtained from the analysis 9 case-control studies (OR = 1.03, 95% CI: 0.99–1.07). The forest plot (Figure 2) showed this result. There was significant heterogeneity in these studies (RCT and cohort study: *I*^2^ = 98%, *P* < 0.01, Figure 2(a); case-control study: *I*^2^ = 72%, *P* < 0.01, Figure 2(b)), so we used random-effects model for analysis, which was consistent with the original model.

### 3.4. Subgroup Analyses and the Meta-Regression

**Figure 3:**
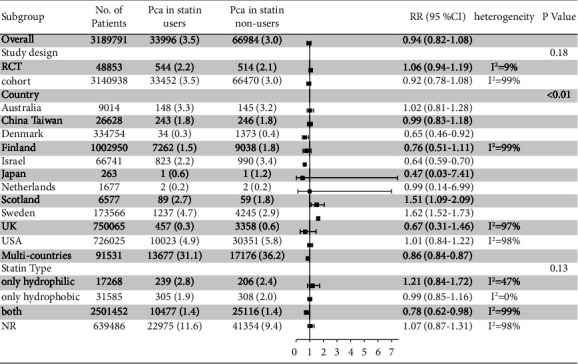
Forest plot of RR for subgroup analysis based on study design, country and statin type.

In order to find the source of heterogeneity, we conducted a subgroup analysis (Figure 3). In the subgroup analysis of the study design, the *P* value for interaction between the two types was 0.18, indicating that the study design did not contribute to the heterogeneity of the literature whereas different countries were one of the sources of heterogeneity because the *P* value for interaction in the subgroup analysis of countries was <0.01. Studies conducted in Denmark (RR = 0.65, 95% CI: 0.46–0.92) and Israel (RR = 0.64, 95% CI: 0.59–0.70) had shown that statins could be associated with decreasing the incidence of PCa. The studies of Scotland (RR = 1.51, 95% CI: 1.09–2.09) and Sweden (RR = 1.62, 95% CI: 1.52–1.73) showed that statins were associated with a higher incidence of PCa. Given that *P* value for interaction in the subgroup analysis of statin type was 0.13, there was no significant difference in the incidence of PCa among different statins.

**Figure 4:**
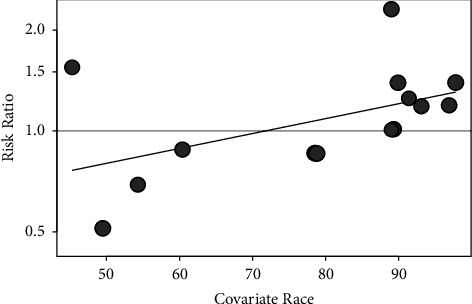
The meta-regression for risk of PCa and race (the percentage of white).

In addition, we used meta-regression analysis for some continuous variables to further find the covariates. The meta-regression models of publication year, follow-up time, age, BMI, population percentage of BMI <30 kg/m^2^, race (the percentage of white), PSA level, GS, and population percentage of cDDD >600 were constructed. For RCT and cohort studies, we found that race (the percentage of whites) (*P* = 0.0430) significantly affected the effects of statins on the incidence rate of PCa (Figure 4). There was no significant relationship between the incidence of PCa and year (*P* = 0.9890), follow-up period (*P* = 0.8267), age (*P* = 0.8852), BMI (*P* = 0.9106), and cDDD <600 (*P* = 0.8718) (Supplementary Material 5). For case-control studies, there was no relationship between the incidence of Pca and the incidence of the year (*P* = 0.8254) and follow-up period (*P* = 0.8254).

### 3.5. Effect of cDDD and Duration of Statins on PCa

**Figure 5:**
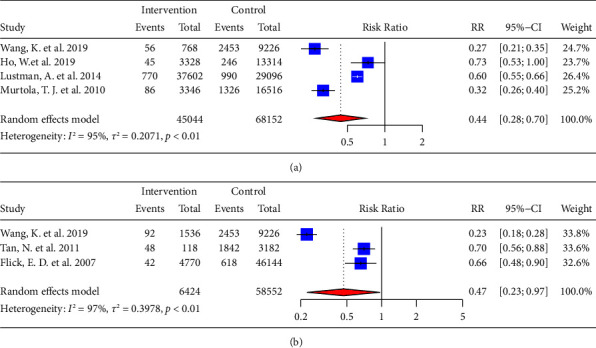
Forest plot of RR for the effect of (a) high cDDD and (b) long duration of statins on PCa.

4 studies recorded the cDDD for statins of each group of participants in detail [32, 38, 40, 48]. And our results showed that statins were associated with a lower risk of PCa (RR = 0.44, 95% CI: 0.28–0.70) when the cumulative defined daily dose (cDDD) was high (cDDD >600) (Figure 5(a)). The results of 3 cohort studies [27, 45, 48] that recorded data from participants who had used statins for long periods showed that using them for more than five years was associated with a decreased incidence of PCa (RR = 0.47, 95% CI: 0.23–0.97) (Figure 5(b)).

### 3.6. Sensitivity Analysis and Publication Bias for Included Studies

**Figure 6:**
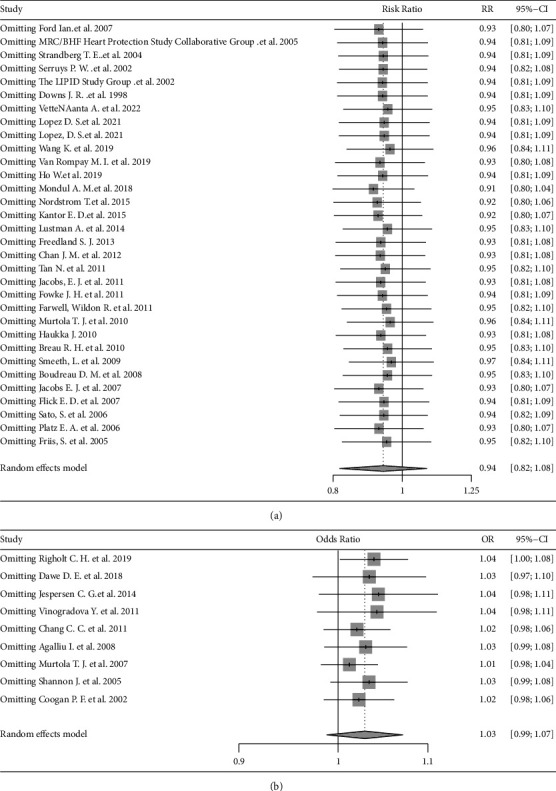
The forest plot for sensitivity analysis. (a) The forest plot for the RR. (b) The forest plot for the OR.

In order to test the stability of our results, we conducted a sensitivity analysis (Figure 6) or cumulative meta-analysis of the overall effect by omitting or adding one study at a time. For RR effect values and OR effect values, omitting some studies had no significant effect on the overall results. Therefore, our results were stable and reliable. For RR effect values, Begg's test (*z* = −0.16, *P* = 0.8712) and Egger's test (*t* = 0.19, *P* = 0.8476) indicated that there was no publication bias [60]. For OR effect values, we also did not determine statistically significant publication bias for Begg's test (*z* = 0.83, *P* = 0.4042) and Egger's test (*t* = 0.78, *P* = 0.4617).

## 4. Discussion

In recent decades, researchers have paid more attention to the anticancer progress of statins. Statins are not only fully explored in PCa [61] but also have a series of active anticancer effects in breast cancer, pancreatic cancer, gastric cancer, colorectal cancer, ovarian cancer, lung cancer, lymphoma, and kidney cancer [62].

Statins work through noncholesterol-mediated or cholesterol-mediated pathways [63]. Statins make cells stay in the G1 or S phase by regulating cyclin and cyclin-dependent kinases. Statins can also activate the molecular mechanism of apoptosis, such as the AKT/FOXO1 pathway in PCa cells [64]. Statins reduce the content of membrane rafts and further changes in cell signal transduction by reducing the content of cholesterol. Finally, the consumption of cholesterol leads to the loss of membrane integrity [65].

In this meta-analysis, we included a total of 41 studies on statin usage and the incidence of PCa, and we used RR and OR as effect values. RR was mainly used in randomized controlled trials or prospective cohort studies to study the effect of statin exposure on the outcome of PCa by designing follow-up studies [66]. The OR value was mainly used in retrospective case-control studies to study the correlation between the case group (PCa patients) and the control group (non-PCa population).

6 RCT studies did not show a significant association between statin use and PCa. These 6 studies explored the relationship between statin use, all-cause mortality, and specific cancer incidence in hypercholesterolemia patients. Cardiovascular events were the main outcome, so there were basic diseases in patients. 10 of 26 cohort studies reported that statins were associated with a lower incidence of PCa, which was in line with our expectations. Only one of nine case-control studies indicated that statins associated with a lower incidence rate of PCa [56]. In addition, a previous meta-analysis showed that statins decreased overall PCa risk [10]. However, the results of two other meta-analyses [9, 11] did not support the hypothesis that statins are associated with the overall risk of PCa, which was consistent with our conclusion.

Many of these studies relied on questionnaires to obtain exposure data and lacked screening adjustments [55]. Nevertheless, the definition of statin exposure varies across studies. Some studies define exposure as the use of statins before PCa diagnosis, which is the most appropriate definition, and some define it as having ever used statins, while others define it as the use of certain statins within a specified period of time. If the definition of exposure includes the condition that patients still use statins after diagnosis with PCa, it may undermine the hypothesis that statins are beneficial to reduce the incidence of PCa. In addition, among these three types of studies, the level of evidence in case-control studies was the lowest [59]. The real results should be more biased towards the combined results of RCTs and cohort studies.

We identified documented cDDD in three cohort studies, and we found that high cDDD (cDDD >600) is significantly associated with a reduced risk of PCa. However, cDDD was the effect value accumulated by the defined daily dose over time, so it was difficult to identify whether long-term duration or a large dose led to the reduction of PCa risk. Then, to explore the impact of duration on the risk of PCa, we recorded and analyzed the specific duration of each group. In 3 cohort studies and 4 case-control studies, some participants used statins for more than 5 years. By analyzing the total RR effect value of these cohort studies and the total OR effect value of these case-control studies, we found that when the duration was more than 5 years, the overall risk of PCa in cohort studies decreased significantly, and the overall risk of PCa in case-control studies decreased but not statistically significant. Long-term use of statins had a more stable effect on the anticancer effect in the body.

It shows high heterogeneity in the overall analysis. In some subgroups, heterogeneity was decreased, indicating that research types and countries were important influencing factors. Researchers could dictate that participants use a certain type of statins in an RCT. However, in observational studies, the type of statins used by participants could not be intervened. In addition, the definition of statin use was different in different studies. Thus, statins type was also one of the sources of heterogeneity.

Meta-regression showed that the proportion of whites was positively correlated with the risk of PCa. On the one hand, this was due to the different genetic susceptibilities of different races. On the other hand, Caucasians were more likely to be diagnosed with PCa because of different levels of medical care in different countries.

Concerning confounding factors, we put forward some thoughts. There was a direct correlation between the malignant degree of PCa and PSA [67]. The higher the malignant degree of the tumor, the higher the serum level of prostaglandin-specific antigen. PSA screening was associated with overdiagnosis of low-risk PCa [47]. Some studies also indicated that serum PSA levels decreased significantly among statin users [68–72]. However, taking aspirin and/or antidiabetic drugs at the same time may change the anticancer effect of statins [27, 41]. Moreover, we speculate that different education levels, income levels, and social status may also be confounding factors for the incidence of PCa.

For the prevention of PCa, we not only pay attention to statins but it is more important that residents maintain good living habits. The goal of the World Cancer Research Fund/American Institute for Cancer Research (WCRF/AICR) Report is to improve health and reduce the global cancer burden through diet, nutrition, and physical activity [73], which is consistent with certain of the purposes of our study. The Cohort of Swedish Men (COSM) revealed that compliance with WCRF/AICR recommendations significantly reduced the overall cancer risk [74]. The treatment of cancer is becoming more complex, and diet, nutrition, and physical activity play an important role as auxiliary means.

Compared with previous meta-analysis, ours included more qualified literature, and the sample size was greatly increased, making the conclusion more universal. Moreover, we not only explored the effects of statin usage on the overall risk of PCa but also further explored the association between cDDD, the duration of statins, and the risk of PCa.

Some limitations should be noted. First, some data were incomplete in different studies. For example, the follow-up period was not recorded in 3 studies, 16 studies lacked age data, 32 studies lacked BMI data, 25 studies lacked race composition data, 33 studies did not record participants' PSA baseline value. And the type of statins in 16 studies was unknown. Second, due to the inclusion of too many studies, it was difficult to unify each study's design methods and the definition of statin exposure, so that the heterogeneity between studies was high. Third, it is difficult to accurately record the long follow-up period and other vague data. Therefore, it remained to be seen whether a certain type of statin and its dosage and duration of use affects the risk of PCa. For future research, more clinical trials and in vitro laboratory experiments are needed to further verify the role of statins in PCa.

## 5. Conclusion

In summary, we conducted a meta-analysis to explore the effects of statin use on the overall incidence of PCa. We found that statins had a neutral effect on the overall risk of PCa, and high cDDD and long duration were associated with a lower risk of PCa, which may contribute to preventing PCa and decreasing its incidence.

## Figures and Tables

**Table 1:** Newcastle-Ottawa scale for assessing the quality of cohort studies in meta-analysis.

No.	Study	Selection				Comparability	Outcome			Score
		Exposed cohort	Nonexposed cohort	Ascertainment of exposure	Outcome not present at start	Comparability of cohorts	Assessment	Follow-up long enough	Adequacy of follow-up	
1	Vettenranta et al.	*∗*	*∗*	*∗*	*∗*	^*∗∗*^	*∗*	*∗*	*∗*	9
2	Lopez et al.	*∗*	*∗*		*∗*	^*∗∗*^	*∗*	*∗*	*∗*	8
3	Lopez et al.	*∗*	*∗*	*∗*	*∗*	*∗*	*∗*	*∗*	*∗*	7
4	Wang et al.	*∗*	*∗*	*∗*	*∗*	^*∗∗*^	*∗*	*∗*	*∗*	9
5	Van Rompay et al.	*∗*	*∗*	*∗*	*∗*	^*∗∗*^	*∗*	*∗*	*∗*	9
6	Ho et al.	*∗*	*∗*	*∗*	*∗*	^*∗∗*^	*∗*	*∗*	*∗*	9
7	Mondul et al.	*∗*	*∗*	*∗*	*∗*	*∗*	*∗*	*∗*	*∗*	7
8	Nordstrom et al.	*∗*	*∗*	*∗*	*∗*	^*∗∗*^	*∗*		*∗*	8
9	Kantor et al.	*∗*	*∗*	*∗*	*∗*	^*∗∗*^	*∗*	*∗*	*∗*	9
10	Lustman et al.	*∗*	*∗*	*∗*	*∗*	^*∗∗*^	*∗*	*∗*	*∗*	9
11	Freedland	*∗*	*∗*		*∗*	^*∗∗*^	*∗*		*∗*	7
12	Chan et al.	*∗*	*∗*	*∗*	*∗*	^*∗∗*^		*∗*	*∗*	8
13	Tan et al.	*∗*	*∗*	*∗*		*∗*		*∗*	*∗*	5
14	Jacobs et al.	*∗*	*∗*		*∗*	*∗*	*∗*	*∗*	*∗*	6
15	Fowke et al.	*∗*	*∗*		*∗*	^*∗∗*^		*∗*	*∗*	7
16	Farwell et al.	*∗*	*∗*	*∗*	*∗*	*∗*	*∗*	*∗*	*∗*	7
17	Murtola et al.	*∗*	*∗*	*∗*	*∗*	*∗*	*∗*	*∗*	*∗*	7
18	Haukka	*∗*	*∗*	*∗*	*∗*	^*∗∗*^	*∗*	*∗*	*∗*	8
19	Breau et al.	*∗*	*∗*	*∗*	*∗*	^*∗∗*^		*∗*	*∗*	7
20	Smeeth et al.	*∗*	*∗*	*∗*	*∗*	*∗*		*∗*	*∗*	6
21	Boudreau et al.	*∗*	*∗*	*∗*	*∗*	^*∗∗*^	*∗*	*∗*	*∗*	8
22	Jacobs et al.	*∗*	*∗*		*∗*	^*∗∗*^		*∗*	*∗*	7
23	Flick et al.	*∗*	*∗*	*∗*	*∗*	^*∗∗*^	*∗*		*∗*	8
24	Sato et al.	*∗*	*∗*	*∗*	*∗*	*∗*	*∗*		*∗*	6
25	Platz et al.	*∗*	*∗*		*∗*	^*∗∗*^	*∗*	*∗*	*∗*	8
26	Friis et al.	*∗*	*∗*	*∗*	*∗*	^*∗∗*^	*∗*	*∗*	*∗*	8

**Table 2:** Newcastle-Ottawa scale for assessing the quality of case-control studies in meta-analysis.

No.	Study	Selection				Comparability	Exposure			Score
		Case definition	Representativeness of cases	Selection of controls	Definition of controls	Comparability of cases and controls	Ascertainment of exposure	Same method of ascertainment	Nonresponse rate	
1	Righolt et al.	*∗*	*∗*	*∗*	*∗*	*∗*		*∗*		6
2	Dawe et al.	*∗*	*∗*	*∗*	*∗*		*∗*	*∗*		6
3	Jespersen et al.	*∗*	*∗*	*∗*	*∗*	*∗∗*	*∗*	*∗*		8
4	Vinogradova et al.	*∗*	*∗*	*∗*	*∗*	*∗∗*	*∗*	*∗*		8
5	Chang et al.		*∗*		*∗*	*∗∗*	*∗*	*∗*	*∗*	7
6	Agalliu et al.		*∗*	*∗*	*∗*	*∗∗*		*∗*		6
7	Murtola et al.	*∗*	*∗*	*∗*	*∗*	*∗∗*	*∗*	*∗*		8
8	Shannon et al.	*∗*	*∗*		*∗*	*∗∗*	*∗*	*∗*		7
9	Coogan et al.	*∗*	*∗*		*∗*	*∗∗*		*∗*	*∗*	7

## Data Availability

All data generated or analyzed during this study are included in this published article and referenced articles are listed in the References section.

## Supplementary Materials

Supplementary Materials Supplementary Material 1: PRISMA 2020 checklist. Supplementary Material 2: Search strategies in this study. Supplementary Material 3: Characteristics of included studies in the meta-analysis and systematic review. Supplementary Material 4: The bias risk map and bias risk summary map in ROB2 excel. Supplementary Material 5: The meta-regression for risk of PCa and year, follow-up period, Age, BMI and cDDD.
