# Supplementary material for: Association of Statin Use with the Risk of Incident Prostate Cancer: A Meta-Analysis and Systematic Review
Source: J Oncol. 2022 Dec 13;2022:7827821. doi: 10.1155/2022/7827821 (PMC9767737; doi:10.1155/2022/7827821)
Supplement: Supplementary Materials — Supplementary Material 1: PRISMA 2020 checklist. Supplementary Material 2: Search strategies in this study. Supplementary Material 3: Characteristics of included studies in the meta-analysis and systematic review. Supplementary Material 4: The bias risk map and bias risk summary map in ROB2 excel. Supplementary Material 5: The meta-regression for risk of PCa and year, follow-up period, Age, BMI and cDDD. [file 7827821.f1.zip › Supplementary Materials 2 (2).docx]

**Supplement 2. Search strategies in this study.**

| Database | Search | Query | Results |
| --- | --- | --- | --- |
| PubMed | #1 | (((((((((((Statin[Title/Abstract]) OR (Atorvastatin[Title/Abstract])) OR (Cerivastatin[Title/Abstract])) OR (Compactin[Title/Abstract])) OR (Fluvastatin[Title/Abstract])) OR (HMG-CoA[Title/Abstract])) OR (Lovastatin[Title/Abstract])) OR (Mevastatin[Title/Abstract])) OR (Pravastatin[Title/Abstract])) OR (Rosuvastatin[Title/Abstract])) OR (Rosvastatin[Title/Abstract])) OR (Simvastatin[Title/Abstract]) | 50325 references |
|  | #2 | ((((((((((((((((Prostatic Neoplasms[Title/Abstract]) OR (Prostate Neoplasms[Title/Abstract])) OR (Neoplasms, Prostate[Title/Abstract])) OR (Neoplasm, Prostate[Title/Abstract])) OR (Prostate Neoplasm[Title/Abstract])) OR (Neoplasms, Prostatic[Title/Abstract])) OR (Neoplasm, Prostatic[Title/Abstract])) OR (Prostatic Neoplasm[Title/Abstract])) OR (Prostate Cancer[Title/Abstract])) OR (Cancer, Prostate[Title/Abstract])) OR (Cancers, Prostate[Title/Abstract])) OR (Prostate Cancers[Title/Abstract])) OR (Cancer of the Prostate[Title/Abstract])) OR (Prostatic Cancer[Title/Abstract])) OR (Cancer, Prostatic[Title/Abstract])) OR (Prostatic Cancers[Title/Abstract])) OR (Cancer of Prostate[Title/Abstract]) | 141411 references |
|  | #3 | #1 AND #2 | 421 references |
| Embase | #1 | (Statin or Atorvastatin or Cerivastatin or Compactin or Fluvastatin or HMG-CoA or Lovastatin or Mevastatin or Pravastatin or Rosuvastatin or Rosvastatin or Simvastatin).ab,kw,ti. | 79781 references |
|  | #2 | (prostate cancer or prostatic cancer or prostate gland cancer or cancer, prostate).ab,kw,ti. | 208188 references |
|  | #3 | #1 AND #2 | 742 references |
| Cochrane | #1 | (Statin): ti,ab,kw or(Atorvastatin):ti,ab,kw or(Cerivastatin):ti,ab,kw or(Compactin):ti,ab,kw or(Fluvastatin):ti,ab,kw or(HMG-CoA):ti,ab,kw or(Lovastatin):ti,ab,kw or(Mevastatin):ti,ab,kw or(Pravastatin):ti,ab,kw or(Rosuvastatin):ti,ab,kw or(Rosvastatin):ti,ab,kw or(Simvastatin):ti,ab,kw | 16052 references |
|  | #2 | (Prostatic Neoplasms):ti,ab,kw or(Prostate Neoplasms):ti,ab,kw or(Neoplasms, Prostate):ti,ab,kw or(Neoplasm, Prostate):ti,ab,kw or(Prostate Neoplasm):ti,ab,kw or(Neoplasms, Prostatic):ti,ab,kw or(Neoplasm, Prostatic):ti,ab,kw or(Prostatic Neoplasm):ti,ab,kw or(Prostate Cancer):ti,ab,kw or(Cancer, Prostate):ti,ab,kw or(Prostate Cancers):ti,ab,kw or(Cancers, Prostate):ti,ab,kw or(Cancer of the Prostate):ti,ab,kw or(Prostatic Cancer):ti,ab,kw or(Cancer, Prostatic):ti,ab,kw or(Cancer of Prostate):ti,ab,kw or(Prostatic Cancers):ti,ab,kw | 15786 references |
|  | #3 | #1 AND #2 | 86 references |
