# Supplementary material for: Association of Statin Use with the Risk of Incident Prostate Cancer: A Meta-Analysis and Systematic Review
Source: J Oncol. 2022 Dec 13;2022:7827821. doi: 10.1155/2022/7827821 (PMC9767737; doi:10.1155/2022/7827821)
Supplement: Supplementary Materials — Supplementary Material 1: PRISMA 2020 checklist. Supplementary Material 2: Search strategies in this study. Supplementary Material 3: Characteristics of included studies in the meta-analysis and systematic review. Supplementary Material 4: The bias risk map and bias risk summary map in ROB2 excel. Supplementary Material 5: The meta-regression for risk of PCa and year, follow-up period, Age, BMI and cDDD. [file 7827821.f1.zip › Supplementary Materials 3 (2).docx]

**Supplementary Materials 3. Characteristics of included studies in the meta-analysis and systematic review.**

| **No.** | **Study** | **Year** | **Country** | **Study design** | **Patient characteristics** | **Definition of statin use** | **Follow up period (years)** | **Age (years)** | **BMI** | **Race** | **PSA**  **(ng/mL)** | **GS** | **type of statins** | **Hydrophilicity** | **No. of patients** | **No. of patients on statins (%)** | **Covariate adjustment** |
| --- | --- | --- | --- | --- | --- | --- | --- | --- | --- | --- | --- | --- | --- | --- | --- | --- | --- |
| 1 | Ford Ian.et al. | 2007 | Scotland | RCT | 6595 men, aged 45 to 64 years, with raised cholesterol levels, were randomized in equal numbers to placebo or pravastatin after initial screening of approximately 81000 subjects in the West of Scotland. | pravastatin (40 mg once daily) | 15 | 55 | NR | NR | NR | NR | pravastatin | only hydrophilic | 6577 | 3291(50.2) | systolic blood pressure, diastolic blood pressure, high-density lipoprotein cholesterol level, low-density lipoprotein cholesterol level, log triglyceride level, nitrate use or nonuse, and presence  or absence of a history of angiNR, diabetes, or hypertension. |
| 2 | MRC/BHF Heart Protection Study Collaborative Group .et al. | 2005 | UK | RCT | Men and women aged about 40 to 80 years with non-fasting blood total cholesterol concentrations of at least  3.5 mmol/L (135 mg/dL) were eligible provided they had a medical history of:  occlusive arterial disease; diabetes mellitus; or treated hypertension (if also male and aged at least 65 years). | to receive 40 mg simvastatin daily | 1994.7-1997.5; 5 | 64 | NR | NR | NR | NR | simvastatin | only hydrophobic | 20536 | 10269 (50.0) | NR |
| 3 | Strandberg T. E..et al. | 2004 | Finland | RCT | patients age 35–70 years with previous myocardial  infarction or angiNR pectoris, serum total cholesterol  5·5–8·0 mmol/L, and serum triglycerides 2·5 mmol/L  or lower. | The initial dose of simvastatin was 20 mg daily,  which was titrated to 40 mg daily in patients who did not reach the target total cholesterol concentrations of  3·0–5·2 mmol/L after 6–18 weeks (37%) by methods  that preserved the double-blind NRture of the study. | 10.4 (9.9-11.3) | NR | NR | NR | NR | NR | simvastatin | only hydrophobic | 4444 | 2221 (50.0) | Adjusted for age, sex, hypertension, smoking, myocardial infarction, and diabetes at baseline. |
| 4 | Serruys P. W. .et al. | 2002 | Netherlands | RCT | A total of 1677 patients (aged 18-80 years) recruited between April 1996 and October 1998 with stable or unstable angiNR or silent ischemia following successful completion of their first PCI who had baseline total cholesterol levels between 135 and 270 mg/dL (3.5-7.0 mmol/L), with fasting triglyceride levels of less than 400 mg/dL (4.5 mmol/L). | fluvastatin, 80 mg/d | 3.9 | (1)60.0 (10.1) ;  (2)60.0 (9.8) | (1)26.7 (3.3);  (2)26.4 (3.3) | NR | NR | NR | fluvastatin | only hydrophilic | 1677 | 844 (50.3) | NR |
| 5 | The LIPID Study Group .et al. | 2002 | Australia | RCT | 4 This randomised placebo-controlled trial was undertaken at 87 centres in Australia and New Zealand, and involved 9014 patients who had had an acute myocardial infarction or a hospital discharge diagnosis of unstable angiNR pectoris 3–36 months before study entry and whose total cholesterol concentration was 4·0–7·0 mmol/L. | pravastatin 40 mg per day | 8 | (1) 62 (55–67) ;  (2) 62 (55–67) | NR | NR | NR | NR | pravastatin | only hydrophilic | 9014 | 4512 (50.1) | NR |
| 6 | Downs J. R. .et al. | 1998 | USA | RCT | included 6605 men and women and was conducted at 2 sites in Texas, Lackland Air Force Base in San Antonio (n = 3737) and Uni厂versity of North Texas Health Science Center in Fort Worth (n = 2868). | Lovastatin (20-40 mg daily) | 5.2 | (1) 58 (7) ; (2)  58 (7) ; | (1)27.1 (3.1); (2)  27.0 (3.0) | 1. White: 89%; Black:   3%; Hispanic:7%;   1. White: 89%; Black:   3%; Hispanic:7%; | NR | NR | Lovastatin | only hydrophobic | 6605 | 3304 (50.0) | NR |
| 7 | VetteNRanta A. et al. | 2022 | Finland | cohort | Finnish Randomized Study of Prostate Cancer Screening in the Tampere or Helsinki districts of Finland. | before PCa diagnosis or by Decem-  ber 31, 2009 | 1996-2015; 17  (11-19) | (1) 59 (55-63);  (2) 59 (55-63) | (1)27.4 (24.3-29.7)  (2) 26.4 (23.9-28.4) | NR | (1) 1.18  (0.68-  2.14);  (2)1.28  (0.74-  2.41) | (1) >=7:  48.0%;  (2) >=7:  38.0% | NR | NR | 30336 | 12059 (39.8) | NR |

| 8 | Lopez D. S.et al. | 2021 | multi- countries | cohort | population based cancer registries from 19 SEER regions, which cover approximately 30% of the population of the US, with Medicare administrative data | use of statin was identified from Part D- NDC file | NR | NR | NR | (1)Non-Hispanic black： 49.7%; Non-Hispanic white：45.3%; Other： 4.96%; (2)Non-Hispanic black：70.3%; Non- Hispanic white：26.4%; Other：3.37%; | NR | NR | NR | NR | 91531 | 44041 (48.1) | Adjusted for age, race/ethnicity,  Charlson Comorbidity Index (CCI), hypogoNRdism, hypertension, diabetes, use of insulin, muscular wasting and disuse atrophy, malaise and fatigue, osteoporosis, erectile dysfunction, depressive disorder, anterior pituitary disorder, education , percentage of adults below poverty line at census tract level, patients’ primary care (PCP), prostate-specific antigen (PSA), and mutual adjustment for TTh and statin. |
| --- | --- | --- | --- | --- | --- | --- | --- | --- | --- | --- | --- | --- | --- | --- | --- | --- | --- |
| 9 | Lopez, D. S.et al. | 2021 | USA | cohort | 89346 NHW, NHB, and Hispanic men  >= 65 years diagnosed with incident PCa (site recode 28010) between 2007  –2011. | use of statin was identifed from Part D- NDC fle (Appendix  Supplemental Table S2). | 5.6 | 74 | NR | NR | NR | NR | NR | NR | 41965 | 11774 (28.1) | age, race/ethnicity, Charlson Comorbidity Index (CCI), hypogoNRdism, hypertension, diabetes, use of insulin, muscular wasting and disuse atrophy, malaise and fatigue, osteoporosis, erectile dysfunction, depressive disorder, anterior pituitary disorder, decreased libido, education, percentage of adults below poverty  line at census tract level, patients’ primary care (PCP), prostate-specifc antigen (PSA), and mutual adjustment for TTh and statin |
| 10 | Wang K. et al. | 2019 | USA | cohort | conducted at a tertiary hospital affiliated to a university in the Southeastern US based on electronic medical records (EMR) data.patients with at least one visit of the urologic clinic because of any prostatic conditions. | Men were treated as statin nonusers up until the time when they were prescribed with statins for the first time, and as users thereafter for the remaining follow‐up time. | 6.6(2.3-11.5) | (1)65(57-72)  (2)61(51-70) | NR | (1) white：1093 (93.1%);  Black：26 (2.2%);  Other:55 (4.7%) ; (2)  white：15064 (91.2%);  Black：103 (1.8%);  Other:388 (7.0%) | NR | (1)>=7:  16.9%  (2)>=7:  23.8% | lipophilic,hydro philic, Both lipophilic and hydrophilic | hydrophilic or hydrophobic | 13065 | 3839 (29.4) | age , race, smoking status, BMI, PCa family history; history of diabetes, cardiovascular disease, chronic kidney disease, hyperlipidemia, hypertension, and benign prostatic disease; medication use of aspirin, angiotensin‐ converting enzyme inhibitor, insulin, vitamin E/multivitamin, fiNRsteride, metformin, testosterone, selenium; PSA level and cumulative times of PSA testing. |
| 11 | Van Rompay M.  I. et al. | 2019 | USA | cohort | The Saskatchewan Ministry of Health compiled de-identified data covered by Saskatchewan Health (SH) and eligible for provincial outpatient prescription drug benefits between January 1, 1990  and December 31, 2014. | among men aged ≥40 years in Saskatchewan | (1)7.0 (7.5)  (2)5.9 (7.0) | (1)59.1  (10.6); (2)58.7  (10.7) | NR | (1)white: 91.4% ; (2)  white: 90.5% | NR | NR | NR | NR | 264820 | 80282 (30.3) | age at index, index year, baseline diabetes, and baseline use of other drugs of interest, |
| 12 | Ho W.et al. | 2019 | ChiNR Taiwan | cohort | men with IHD and aged between 55 and 100 were acquired from the NRtioNRl Health Insurance Research Databaser in Taiwan | having at least one statin prescribed during the follow-up period. | 2001.01.01 to  2008.12.31; 6.32 | (1)68.0 (7.6)  (2)68.0 (8.1) | NR | NR | NR | NR | Simvastatin, Fluvastatin, Atorvastatin, Lovastatin, Rosuvastatin, Pravastatin | hydrophilic or hydrophobic | 26628 | 13314 (50.0) | Adjusted by comorbidities, comedication, number of outpatient visits, number of hospitalizations. |
| 13 | Mondul A. M.et al. | 2018 | USA | cohort | aged 45-64 years who were recruited from four US communities – Forsyth County, North CaroliNR; suburban Minneapolis, Minnesota; Washington County, Maryland; and Jackson, Mississippi. | use of lipid-lowering drugs was ascertained from information collected at each study visit through visit 4 (1996-1998) and from information collected during the follow-up telephone calls beginning in 2006 through the end of follow-up. | NR | (1)64; (2)63 | (1) 29; (2) 28.4 | (1) white: 96.9%; black:1.6%; other:1.4%; (2) white: 97.8%; black:1.0%; other:1.2%; | NR | NR | NR | NR | 4887 | 953 (19.5) | Adjusted for age, joint categories of race and field center, height, updated BMI, updated cigarette smoking status, updated diabetes status, updated aspirin use, and education level. |
| 14 | Nordstrom T.et al. | 2015 | Sweden | cohort | 185,667 men having a first recorded PSA test and 18,574 men having a first prostate biopsy in Stockholm County, Sweden for the period 2007–2012. | Drug use was defined as any dispensed prescription of the drug within two years before biopsy. | NR | (1)64.1 (10.7)  (2)54.0(12.1) | NR | (1) African American: 10.2%; White:89.3%;  Other: 0.5%; (2)  African American: 10.8%; White:88.2%;  Other: 0.9%; | (1)1.2  (1.91);  (2)0.98  (1.2) | (1)>=7:  29%;  (2)>=7:23  % | Hydrophilic or Hydrophobic | hydrophilic or hydrophobic | 173566 | 26432 (15.2) | adjusted for age, log-transformed PSA level, PSA quotient, comorbidity, educatioNRl level and medication use. |
| 15 | Kantor E. D.et al. | 2015 | USA | cohort | from 2002–2009 in 12 southeastern states,  including: Alabama, Arkansas, Florida, Georgia, Ken tucky, LouisiaNR, Mississippi, North CaroliNR, South CaroliNR, Tennessee, Virginia, and West Virginia. English-speaking  persons between the ages of 40 and 79. | Statin use was ascertained by a series of questions  about history of high cholesterol and current use of cholesterol-lowering  medications. | 2002–2009 and  2010.12.31 | NR | NR | (1)white: 54.3%; black:  2.5%; other:0.2%; missing:43%; (2)white: 53.1%; black: 4.1%;  other:0.3%; missing:42.5%; | NR | NR | NR | NR | 32091 | 4503 (14.0) | Adjusted for age, race/ethnicity, enrollment source, household income, insurance coverage, time since last doctor visit, history of  prostate specific-antigen screening, history of digital rectal exam, history of high cholesterol, and family history of  prostate cancer. |

| 16 | Lustman A. et al. | 2014 | Israel | cohort | male individuals who were enrolled with the Central Region of Clalit Health Services for the period from 1 January 2001 to 31 December 2009,  aged 45–85 years. | From 1  January 2000 until 31 December 2000 statin use was recorded | 2001.01.01 to  2009.12.31 | (1)59.3 (59.2–  59.4)(2)58.1 (58–  58.2) | (1)31.3 (31.2–  31.4); (2)29.2  (29.1–29.3) | NR | NR | NR | Simvastatin,Lo vastatin, Atorvastatin, Fluvastatin Rosuvastatin, Pravastatin | hydrophilic or hydrophobic | 66741 | 37645 (56.4) | adjusted for age, socioeconomic factors immigrant and economic status, DM, BMI, CVD and smoker. |
| --- | --- | --- | --- | --- | --- | --- | --- | --- | --- | --- | --- | --- | --- | --- | --- | --- | --- |
| 17 | Freedland S. J. | 2013 | USA | cohort | Eligible men were aged 50–75 years, with a serum PSA of 2.5–10 ng/ml. | Subjects were asked all medica- tions they used, including statins, which were entered into the case report forms. | 4 | (1) 63.3 (6.0) ;  (2) 62.6 (6.0) | (1)27.4 (25.4–  29.9); (2) 26.7  (24.7–29.2) | NR | (1) 5.7  (1.9) ;  (2)6.0  (1.9) | NR | NR | NR | 6729 | 1174 (17.4) | Adjusted for age, body mass index, race, PSA, digital rectal examiNRtion findings, transrectal ultrasound prostate volume, study treatment, coroNRry artery disease, diabetes, hypertension, smoking, family history, alcohol intake, baseline testosterone and baseline dihydrotestosterone levels. |
| 18 | Chan J. M. et al. | 2012 | USA | cohort | age 65 or older and living in 6 geographic regions of the United States in 2000 to 2002:  Birmingham, Alabama; Minneapolis, Minnesota; Palo  Alto, California; Pittsburgh,  Pennsylvania; Portland, Oregon; and San Diego, California. | as any use in the previous two weeks | 7 | (1)73.1 (5.5)  (2)73.5 (5.9) | (1)27.7 (3.7);  (2)27.3 (3.9) | NR | NR | NR | NR | NR | 5069 | 1377 (27.2) | race, BMI, marital status, family history of prostate cancer, number of comorbidities, physical activity, and smoking history |
| 19 | Tan N. et al. | 2011 | USA | cohort | men who underwent prostate biopsy from 2000 to 2007 at Cleveland Clinic. | Statin use was determined using outpatient pharmacy records | 2000-2007 | (1)65.7 (60–71);  (2)63.5 (57–69) | NR | NR | (1)5.13  (3.38–  8.53);  (2)5.98  (4.47–  8 93) | (1)>=7:61  .4%;  (2)>=7:72  .4% | NR | NR | 4204 | 1022 (24.3) | age, body mass index, African- American race, number of cores taken and prostate volume |
| 20 | Jacobs, E. J. et al. | 2011 | USA | cohort | participants in the CPS-II Nutrition Cohort, a prospective  study of cancer incidence in the United States established in 1992 and described in detail elsewhere. | provided as examples the brand NRmes for 4 statins commonly used at that time  (lovastatin, pravastatin, simvastatin, and fluvastatin) | 1997-2007 | NR | NR | NR | NR | NR | lovastatin, pravastatin, simvastatin, fluvastatin | hydrophilic or hydrophobic | 47595 | 2252 (4.7) | age, sex, race, education, smoking, BMI, physical activity level, NSAID use, hormone therapy, history of elevated cholesterol, heart disease, diabetes, and hypertension. |
| 21 | Fowke J. H. et al. | 2011 | USA | cohort | Men scheduled for a diagnostic prostate biopsy between 2002 and 2010 at a Vanderbilt University Medical Center (NRshville, TN), the Tennessee Valley Veteran’s Admin 厂istration Hospital (NRshville, TN), or Urology Associates, a private urology clinic in NRshville, were approached for recruitment. Eligible participants were 40 years of age or older and had no prior prostate cancer diagnosis. | Statin use included simvastatin, atorvastatin, lovastatin, , rosuvastatin, pravastatin, and fluvastatin. | 2002-2010 | NR | NR | (1)white: 97.8%; black:  0.8%; other or  unknown: 1.4%;  (2)white: 97.8%; black:  0.9%; other or  unknown: 1.2%; | (1) 6.1 ;  (2) 6.8 | (1)>=7:  19.0%;  (2)>=7:  18.7% | Simvastatin, Atorvastatin | hydrophilic or hydrophobic | 2148 | 783 (36.5) | age, race, family history, BMI, WHR, height, aspirin use, and treatment for CVD, diabetes, or BPH. |
| 22 | Farwell, Wildon R. et al. | 2011 | USA | cohort | The electronic and administrative files of a large cohort of men taking a statin or antihypertensive medication were obtained from the Veterans Affairs New England Healthcare System.  Prostate cancer incidence among these two patient populations was compared. | Statin users were defined as patients who filled prescriptions for any of the following medications: atorvastatin, fluvastatin, lovastatin, pravastatin, or simvastatin. | 5.6(2.0-11) | (1)66.3 (10.4);  (2)66.3 (10.4) | NR | (1)Non-Hispanic White:60.4; Black/African American: 8.3%; Hispanic:13.2% Asian:7.6% Other: 10.4%; (2)Non-Hispanic White:63.5%; Black/African American: 6.9%; Hispanic:13.4%  Asian:7.9% Other: 8.4% | NR | (1)>=7:45  .1%;  (2)>=7:52  %; | atorvastatin, fluvastatin, lovastatin, pravastatin, simvastatin. | hydrophilic or hydrophobic | 55884 | 41087 (73.5) | statin use (yes or no), fiNRsteride use history (yes or no), age (years), serum total cholesterol (mg/dL), race (white, black, other, or missing), smoking history (yes or no), aspirin use (yes or no), heart disease (yes or no), diabetes mellitus (yes or no), history of prostate- specific antigen test (yes or no). |
| 23 | Murtola T. J. et al. | 2010 | Finland | cohort | men aged 55–67 years and residing in the metropolitan areas of Helsinki and Tampere were identified from the population register of Finland. | Each man with no medication reimbursements at baseline was treated as a current non-user until the first medication purchase, after which the status changed to a current user. | (1)6.92 (0.0–9.00);  (2)6.92 (0.0–8.92) | NR | (1) 26.8 (2)26.0 | NR | (1)1.05  (2)1.10 | NR | Atorvastatin, Fluvastatin, Simvastatin | hydrophilic or hydrophobic | 23208 | 6692 (28.8) | age, family history of prostate cancer, use of aspirin, antidiabetic drugs and/or antihypertensive drugs, number of PSA screens and calendar period of screening. |

| 24 | Haukka J. | 2010 | Finland | cohort | The study population of statin users included all individuals residing in Finland who (1) have purchased at least one prescription of any statin between January 1, 1996 and December 31, 2005, and had no cancer diagnosis at the date of the first purchase. | have purchased at least one prescription of any statin. The following substances were included: simvastatin (ATC code C10AA01), lovastatin (C10AA02), pravastatin (C10AA03), fluvastatin (C10AA04), atorvastatin (C10AA05), cerivastatin (C10AA06), and rosuvastatin (C10AA07). | 1996.1.1-  2005.12.31; 8.8  yeas | 60 | NR | NR | NR | NR | Simvastatin, Atorvastatin, Fluvastatin, Pravastatin, Rosuvastatin | hydrophilic or hydrophobic | 944,962 | 472481 (50.0) | age, group (statin user on nonuser), and follow-up period using Poisson regression. |
| --- | --- | --- | --- | --- | --- | --- | --- | --- | --- | --- | --- | --- | --- | --- | --- | --- | --- |
| 25 | Breau R. H. et al. | 2010 | USA | cohort | men residing in Olmsted County, Minnesota. | At baseline each participant reported all prescribed and over-the-counter medications taken on a daily basis, and  the date at which the medication was first taken. | (1)15.7(15.1-16.5);  (2)15.2(13.1-16.2) | NR | NR | NR | NR | NR | NR | NR | 2447 | 634 (25.9) | age, diabetes, hypertension, coroNRry heart disease, NSAID use, 5-α reductase inhibitor use, and α-blocker use |
| 26 | Smeeth, L. et al. | 2009 | UK | cohort | The source population was all patients registered with a general practice contributing to the THIN database between January 1995 and December 2006, comprising the electronic medical records for 5.5 million patients derived from 303 general practices. | All patients aged 40–80 years who received their first prescription for a statin on or after 1 January 1995 and with  >12 months prior continuous registration with a general practice contributing to the database were included in the study. | 1995.1-2006.12;  4.4 | NR | NR | NR | NR | NR | Rosuvastatin, Simvastatin, Atorvastatin, Cerivastatin | hydrophilic or hydrophobic | 729529 | 129288 (17.7) | age, sex, propensity score, year of index date, first diagnosis of any of the following post-index date: diabetes, cerebrovascular disease, coroNRry heart disease, peripheral vascular disease, other atheroma, atrial fibrillation, heart failure, hyperlipidaemia, hypertension, other circulatory disease, cancer, dementia, first use of any of the following post- index date: aspirin, nitrates, fibrates, b- blockers, calcium channel blockers, potassium channel activators, diuretics, positive inotropes, anticoagulants, antihypertensives, or other cardiovascular drugs. |
| 27 | Boudreau D. M. et al. | 2008 | USA | cohort | (1) continuously enrolled in Group Health’s integrated group practice for  at least two years  during the study period of 1 January 1990 to 31 August 2005; (2) between the age of 45–79 years anytime during the study period; (3) residing in 1 of 13 Washington  counties covered by the western Washington Surveillance, Epidemiology, and End Results (SEER) cancer registry,  and (4) no prior history of prostate cancer as identified in  the SEER registry. | men with 2+ dispensings for a statin within any six-month period and who used statins  for at least one year. Men with 1 or less statin dispensing or  less than one year of statin use were considered non-users. We considered men current users if statins were used within the previous 12 months and past users if statins  were used more than 12 months prior. | 1990.1.1--  2005.8.31; 5.7 | (1)57.6(7.6)  (2)57.4(9.3) | NR | NR | NR | NR | Hydrophobic or Hydrophilic | hydrophilic or hydrophobic | 83372 | 12013 (14.4) | age, diabetes, hypercholesterolemia, other lipid lowering drug use, and NSAID use |
| 28 | Jacobs E. J. et al. | 2007 | USA | cohort | male participants in the Cancer Prevention Study-II Nutrition Cohort, a prospective study of cancer incidence and mortality in the United States established in 1992. | questionNRire asked participants to report whether they had taken any ‘‘cholesterol- lowering  drugs’’ regularly during the past year, and provided as examples the brand NRmes for four statins commonly used at that time. | 1997-2003 | NR | NR | (1)white: 97.8%; black:  0.8%; other or  unknown: 1.4%;  (2)white: 97.8%; black:  0.9%; other or  unknown: 1.2%; | NR | NR | NR | NR | 55454 | 13582 (24.5) | age, historyof prostate-specific antigen testing, and other potential prostate cancer risk factors |
| 29 | Flick E. D. et al. | 2007 | USA | cohort | the cohort consists of 84,170 Northern and Southern California Kaiser Permanente (KP) members  who completed mailed questionNRires in 2002 to 2003 | Exposure to statins was defined as more than a total  of 100 days supply of one or more statins dispensed (henceforth referred to as ‘‘ever use’’). | 1991-2004; 8.8 | NR | NR | (1)Non-Hispanic White:60.4; Black/African American: 8.3%; Hispanic:13.2% Asian:7.6% Other: 10.4%; (2)Non-Hispanic White:63.5%; Black/African American: 6.9%; Hispanic:13.4%  Asian:7.9% Other: 8.4% | NR | NR | NR | NR | 69047 | 22903 (33.2) | race, diabetes, and Kaiser Permanente California region |

| 30 | Sato, S. et al. | 2006 | Japan | cohort | The subjects were 263 patients with coroNRry heart disease who were from Osaka prefecture and who were admitted to the Osaka Medical Center for Cancer and Cardiovascular Diseases between September 28, 1991  and March 31, 1995. | Pravastatin medicated' refers to patients in Groups I and IIa who took pravastatin for >= 75 % of the study period, and patients in Groups IIb and III who took pravastatin for >25% of the study period | 1991.9.28-  1995.3.31 | NR | NR | NR | NR | NR | NR | NR | 263 | 179 (68.1) | NR |
| --- | --- | --- | --- | --- | --- | --- | --- | --- | --- | --- | --- | --- | --- | --- | --- | --- | --- |
| 31 | Platz E. A. et al. | 2006 | USA | cohort | Participants were members of the Health ProfessioNRls  Follow-up Study, an ongoing prospective cohort study of diet  and other risk factors for heart disease,  cancer, and other condi厂tions | The use of any cholesterol- lowering drug, including the other cholesterol-lowering drugs reported in 2000, was counted as statin use. | 1990.2.1-2002.1.31 | (1) 63.2 ( 8.7);  (2)60.9 (9.3) | (1)26.3 (3.5);  (2)25.9 (3.5) | (1)White race: 89.9%; (2)White race:91.2% | NR | NR | NR | NR | 30643 | 2847 (9.3) | age; body mass index at age 21; height; pack-years  of cigarette smoking in the previous decade; fi rst-degree family history of  pros 厂tate cancer; major ancestry; diabetes mellitus; vasectomy; vigorous physical ac 厂tivity; use of aspirin; intakes of total energy, calcium, fructose, α -linolenic acid, tomato sauce, red meat, fi sh, and alcohol; intake of supplemental zinc; and high intake of vitamin E |
| 32 | Friis, S. et al. | 2005 | Denmark | cohort | From the files of the CPR, we identified 343,959 individuals in North Jutland County who were 30 – 80 years of age during the period 1 January 1989 to 31 December 2002 and resident in the county on 1 January 1989. | individuals in the study population who received at least 2 prescriptions for statins(atorvastatin, cerivastatin, fluvastatin, lovastatin, pravastatin and simvastatin) between 1 January 1989 and 31 December 2002 and who were free of cancer at date of second prescription. | 1989-2002; 3.3  (0-14) | (1)60.7; (2) NR | NR | NR | NR | NR | atorvastatin, cerivastatin, fluvastatin, lovastatin, pravastatin, simvastatin | hydrophilic or hydrophobic | 334754 | 12251 (3.7) | NR |
| 33 | Righolt C. H. et al. | 2019 | CaNRda | case control | men 40 years or older who were registered with MH during 2000 to 2014 with >=5 years of insur厂ance coverage | Using the DPIN, we measured all statin use before the index date (the case's diagnosis date). | 2000-2014 | NR | NR | NR | NR | NR | Atorvastatinb, Cerivastatin, Fluvastatin, Lovastatin, Pravastatin, Rosuvastatin, Simvastatin | hydrophilic or hydrophobic | 56133 | 14093 (25.1) | the matching variables (age, regioNRl health authority of residence, and length of drug use coverage), income quintile, number of physician visits in the 5-year period before the index date, screening indicator, chronic cardiovascular disease (excluding hypertension), diabetes, and ever use of non-statin lipid 厂lowering drugs, metformin, other oral hypoglycemic drugs, insulin, aspirin, and non-aspirin NSAIDs. |
| 34 | Dawe D. E. et al. | 2018 | CaNRda | case control | The source population consisted of all men aged 40 years or older registered with the SPDP 1990-2010, with no prior history of cancer (except for non- melanoma skin cancer). | Detailed histories of use of prescribed statins (Table 1) and 18 other drug classes were obtained from the SPDP for the period between the index date and January 1, 1976, or the coverage.  initiation date, whichever was later. | 1990-2010 | NR | NR | NR | NR | NR | Hydrophobic only or Hydrophilic only | hydrophilic or hydrophobic | 63724 | 10020 (15.7) | only for matching |
| 35 | Jespersen C. G.et al. | 2014 | Denmark | case control | This case-control study was conducted in Denmark. | Current statin use was defined as at least one redeemed prescription 0–12 months before the index date, but those redeeming their first statin prescription within 6 months prior to the index date were categorized as nonusers. Study subjects who redeemed atleast one statin prescription during the study period, but who did not redeem any statin  prescriptions within one year prior to the index date, were classified as former statin users. | 1997-2010 | 72 | NR | NR | NR | NR | hydrophilic only or hydrophobic only | hydrophilic or hydrophobic | 254880 | 47299 (18.6) | age, level of comorbidity, use of aspirin and non-aspirin NSAID use, and level of education. |
| 36 | Vinogradova Y. et al. | 2011 | UK | case control | The QResearch database is one of the largest general practice databases containing anonymised clinical records for over 11 million patients  registered with 574 UK general  practices. | we recorded the use of a medication as “ever used” | 1998.1.1-2008.7.1 | 69 | NR | (1)White: 90%; black:10%; (2)White: 89%; black:11% | NR | >=7:  68.6% | Atorvastatin, Pravastatin, Simvastatin | hydrophilic or hydrophobic | 76617 | 14282 (18.6) | Townsend quintile, body mass index, smoking status, myocardial infarction, coroNRry heart disease, diabetes, hypertension, stroke, rheumatoid arthritis, use of NSAIDs, Cox2- inhibitors, aspirin |

| 37 | Chang C. C. et al. | 2011 | ChiNR Taiwan | case control | Cases consisted of all patients who were aged 50 years and older and had a first-time diag厂nosis of prostate cancer for the period between 2005 and 2008. The controls were matched to cases by age, sex, and index date. | a patient as a statin user if they had at least 2  prescriptions in the 60-month period (or the 98-month period for the 10-year aNRlysis). | 2005.1.1-  2008.12.31 | (1)71.98 (9.43);  (2)71.95 (9.41) | NR | NR | NR | NR | NR | NR | 1940 | 322 (16.6) | matching variable, diabetes, hypertension, CHD, BPH, use of NSAIDs, use of other lipid-lowering drugs, number of physician visits, and number of hospitalizations. |
| --- | --- | --- | --- | --- | --- | --- | --- | --- | --- | --- | --- | --- | --- | --- | --- | --- | --- |
| 38 | Coogan P. F. et al. | 2010 | USA | case control | patients admitted to participating hospitals in New York, Philadelphia, and Baltimore from 1992 to 2008. The population base for the study comprised people living within 50 miles of a participating hospital. | Information on all statin prescription were extracted from the NHRI prescription database. | 1992-2008 | (1) 62; (2)54 | NR | NR | NR | NR | NR | NR | 3374 | 526 (15.6) | 5-year age categories, study center, interview year, body mass index, alcohol use, pack-years of smoking, race, family history of prostate cancer, number of doctor visits made 2 years before hospital admission, education, and NSAID use. |
| 39 | Agalliu I. et al. | 2008 | USA | case control | Subjects were Caucasian and African- American men re 厂siding in King County, Washington, aged 35–74 years | Regular statin use was defined as use at least four  times/week for at least three continuous months, beginning 12 or more months before hospital admission. | 2002.1.1-  2005.12.31 | NR | NR | NR | NR | NR | Hydrophobic only or Hydrophilic only | hydrophilic or hydrophobic | 1943 | 265 (13.6) | age, race, and prostate cancer screening within the 5-year period before the reference date. |
| 40 | Murtola T. J. et al. | 2007 | Finland | case control | All newly diagnosed prostate cancer cases in Finland during 1995 to 2002 and matched controls (24,723 case control pairs) were identified from the Finnish Cancer Registry and the Population Register Center, respectively. | Ever use: use at least once  a week for 3 months or longer.  Current use:  use within the year prior to the reference date. | 1995-2002 | 68 | NR | (1)Caucasian:84.2%; African American:15.8%; (2)Caucasian:89.6%; African American:10.4% | NR | (1)>=7:  47.1%;  (2)none | Atorvastatin, Fluvastatin, Lovastatin, Pravastatin, Simvastatin | hydrophilic or hydrophobic | 49446 | 5061 (10.2) | age, usage of diuretics, calcium channel blockers, angiotensin- converting enzyme inhibitors, angiotensin receptor blockers, metformin, sulfonylureas, and human insulin. |
| 41 | Shannon J. et al. | 2005 | USA | case control | All veterans referred to the PVAMC  urol厂ogy clinic for a prostate biopsy between December 2001 and August 2004 were eligible. | atorvastatin since 1998,  cerivastatin from 1999 to 2001,  fluvastatin since 1996, lovastatin, pravastatin, and simvastatin | 1997.5-2004.8 | NR | NR | 91 | NR | NR | NR | NR | 302 | 133 (44.0) | age, race, body mass index, nonsteroidal antiinflammatory drug use, diabetes, total caloric intake, and use of other lipid-lowering drugs. |
| 42 | Coogan P. F. et al. | 2002 | USA | case control | patients with cancer and other diagnoses admitted to hospitals in Massachusetts, New York, Baltimore, and Philadelphia in the Case-Control Surveillance Study. | Subjects were considered to have used statins if they had been prescribed a statin drug for at least 3 months prior to enrollment. | 1987-2001 | NR | NR | 1. White 91.0%; Black   8.0%; Other 1.0%;   1. White 93.6%; Black   2.5%; Other 4.0%; | NR | NR | Lovastatin, Simvastatin, Pravastatin | hydrophilic or hydrophobic | 2396 | 189 (7.9) | age, year of interview, study center, education, number of doctor visits 2 years before hospitalization, religion, race, alcohol consumption, and body mass index. |
