# Supplementary material for: Association of Statin Use with the Risk of Incident Prostate Cancer: A Meta-Analysis and Systematic Review
Source: J Oncol. 2022 Dec 13;2022:7827821. doi: 10.1155/2022/7827821 (PMC9767737; doi:10.1155/2022/7827821)
Supplement: Supplementary Materials — Supplementary Material 1: PRISMA 2020 checklist. Supplementary Material 2: Search strategies in this study. Supplementary Material 3: Characteristics of included studies in the meta-analysis and systematic review. Supplementary Material 4: The bias risk map and bias risk summary map in ROB2 excel. Supplementary Material 5: The meta-regression for risk of PCa and year, follow-up period, Age, BMI and cDDD. [file 7827821.f1.zip › Supplementary Materials 4.pdf]

A

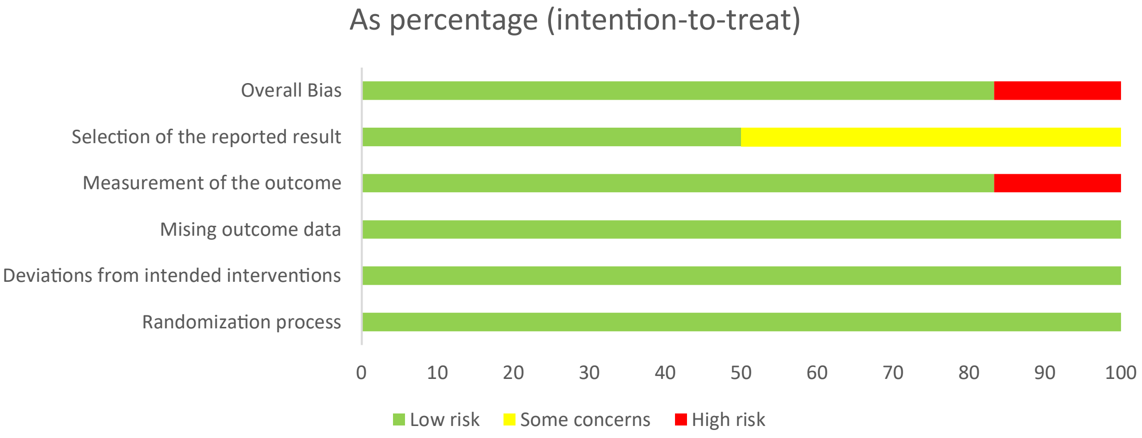

B

Intention-to-treat	Unique ID	Study ID	Experimental	Comparator	Outcome	Weight	D1	D2	D3	D4	D5	Overall
	2007	WOSCOPS	pravastatin	placebo	The primary cause of death	1	+	+	+	+	!	+
	2005	ISRCTN48489393	simvastatin	placebo	all vascular deaths and of all non-vascular deaths	1	+	+	+	+	!	+
	2004	4S	Simvastatin	placebo	all-cause mortality	1	+	+	+	+	+	+
	2002-1	LIPS	fluvastatin	placebo	Survival time free of MACE	1	+	+	+	+	+	+
	2002-2	LIPID	pravastatin	placebo	all-cause mortality	1	+	+	+	-	+	-
	1998	AFCAPS / TexCAPS	Lovastatin	placebo	the incidence of prostate cancer	1	+	+	+	+	!	+

+ Low risk

! Some concerns

\- High risk

D1 Randomisation process

D2 Deviations from the intended interventions

D3 Missing outcome data

D4 Measurement of the outcome

D5 Selection of the reported result
