# Supplementary figures and images for: Association of Statin Use with the Risk of Incident Prostate Cancer: A Meta-Analysis and Systematic Review
Source: J Oncol. 2022 Dec 13;2022:7827821. doi: 10.1155/2022/7827821 (PMC9767737; doi:10.1155/2022/7827821)

A

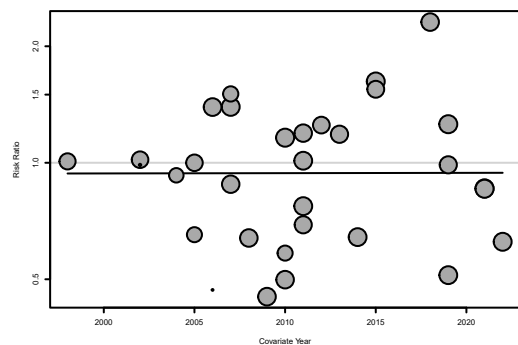

B

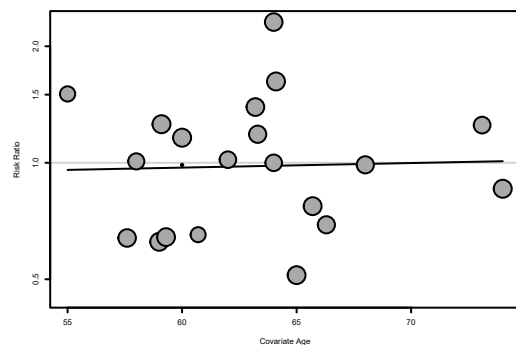

C

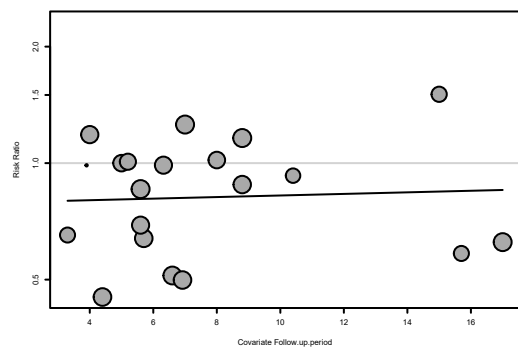

D

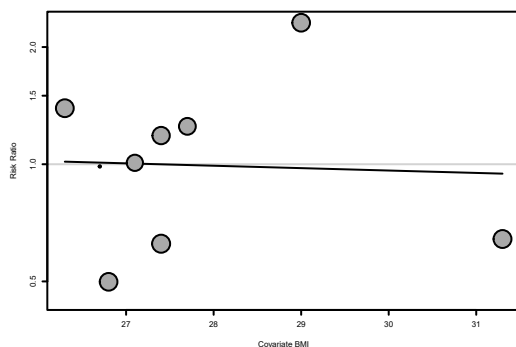

E

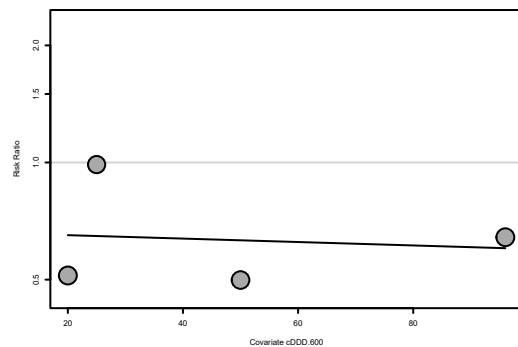

Supplement: Supplementary Materials — Supplementary Material 1: PRISMA 2020 checklist. Supplementary Material 2: Search strategies in this study. Supplementary Material 3: Characteristics of included studies in the meta-analysis and systematic review. Supplementary Material 4: The bias risk map and bias risk summary map in ROB2 excel. Supplementary Material 5: The meta-regression for risk of PCa and year, follow-up period, Age, BMI and cDDD. [file 7827821.f1.zip › Supplementary Materials 5.pdf]
